# Libman-Sacks endocarditis in patients with systemic lupus erythematosus with secondary antiphospholipid syndrome

**DOI:** 10.22088/cjim.10.3.339

**Published:** 2019

**Authors:** Yousef Mohammadi Kebar, Leili Avesta, Afshin Habibzadeh, Mehdi Hemmati

**Affiliations:** 1 Department of Internal Medicine, Ardabil University of Medical Sciences, Ardabil, Iran; 2 Department of Cardiology, Ardabil University of Medical Sciences, Ardabil, Iran; 3 Department of Medicine, Medstar Health (Baltimore)/ Medstar Georgetown University Hospital, Washington, District of Columbia, USA

**Keywords:** Systemic lupus erythematosus, Antiphospholipid syndrome, Libman-Sacks endocarditis

## Abstract

**Background:**

Libman-Sacks endocarditis (LSE) is characterized by sterile lesions that commonly affect the aortic and mitral heart valves. Antiphospholipid syndrome (APS) and systemic lupus erythematosus (SLE) have been associated with LSE. Cardiac manifestations including LSE could be interrelated with other manifestations and early diagnosis could help in preventing further complications.

**Case presentation:**

Here, we report three cases of LSE in SLE patients with secondary APS. All patients presented with neurological manifestations and LSE was diagnosed by Transesophageal echocardiography (TEE). All three patients were treated for the underlying disease and also received anticoagulant therapy.

**Conclusion:**

In all patients with SLE and secondary APS, LSE should be considered if a patient manifests any evidence of neurologic involvement.

LSE is a sterile and verrucous vegetation around the heart valves (1-3). LSE is usually associated with autoimmune diseases especially SLE and APS (1, 2). LSE mostly affects mitral and aortic heart valves (2), which may cause thromboembolic cerebrovascular events, valvular regurgitation, and increased risk of infective endocarditis (2-4). Although APS and valvular involvements are not rare, and have mostly low clinical significance, they could cause severe complications. Secondary APS compared to primary APS also have higher rate of cardiac involvement, mostly due to the autoimmune causes related to the SLE (4). Here, we report three cases of LSE in patients with SLE and secondary APS diagnosed after presenting with neurological manifestations. 

## Case presentation

**Case 1: **A 39-year-old female with a history of stroke 7 months prior to admission, presented with right sided hemiparesis and aphasia. Her home medications include clopidogrel 75 mg daily, aspirin 81 mg daily and losartan 25 mg daily. She had consulted the rheumatology department due to complaints of periodic polyarthralgia and positive anticardiolipin antibody (Ab), lupus anticoagulant, beta2-glycoprotein Ab, antinuclear antibody (ANA), anti-dsDNA and low complement levels. Patient was admitted with the diagnosis of SLE and APS. Transesophageal echocardiography (TEE) showed bicuspid aortic valve leaflets with mild aortic stenosis (AS) and mild aortic insufficiency (AI) with two nonmobile small masses (5x5 mm and 9x5 mm) on the tip of right cusp and non-coronary cusp suggestive of non-bacterial thrombotic endocarditis (Libman-Sacks endocarditis) (figure 1a and b).

**Figure 1.a:**
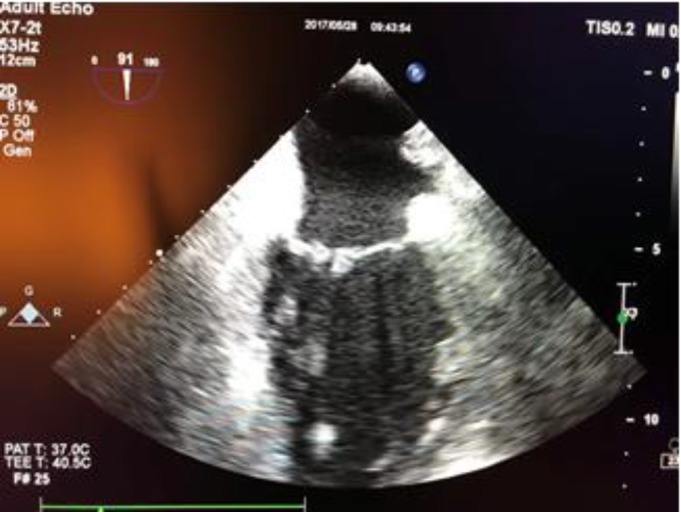
Transesophageal echocardiography (TEE) showed two small vegetations on the tip of right cusp and non-coronary cusp of bicuspid aortic valve

**Figure 1b:**
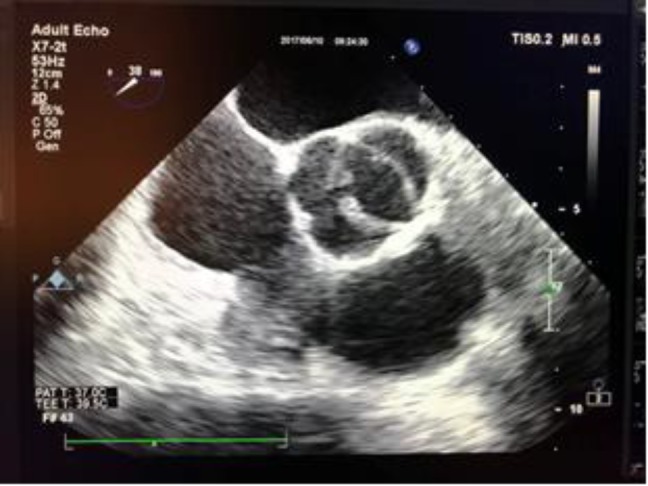
Transesophageal echocardiography (TEE) showed two small vegetations on the tip of right cusp and non-coronary cusp of bicuspid aortic valve

She was started on intravenous (IV) heparin and bridged to oral warfarin to achieve an international normalized ratio (INR) goal of 2-3. The patient was also treated with prednisolone 20 mg, azathioprine 50 mg three times daily and hydroxychloroquine 200 mg twice daily. The patient was stable during her hospital stay. She was discharged with warfarin 5 mg daily with INR goal of 2-3, prednisolone 7.5 mg daily, azathioprine 50 mg three times daily and hydroxychloroquine 200 mg twice daily. At 1 year follow-up, she was symptom free without any SLE flair-up or cardiovascular incident. Follow-up TEE was indicative of no vegetations. 

**Figure 2:**
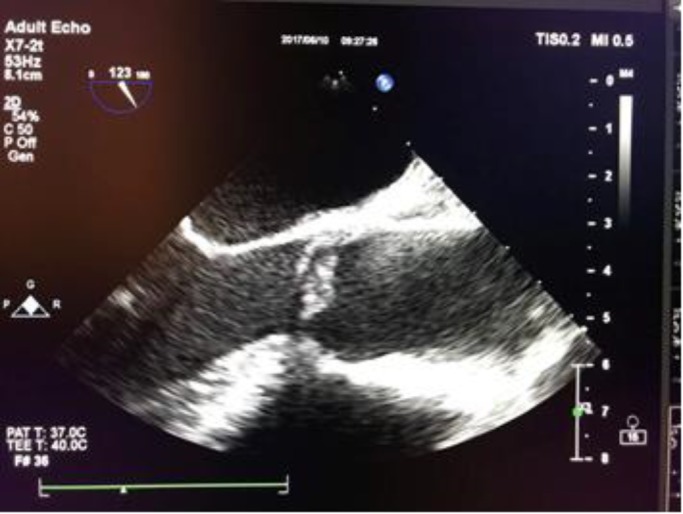
Transesophageal echocardiography (TEE) revealed a thickened mitral valve with two small vegetations on the tip of anterior (and posterior mitral valve leaflets

**Case 2: **A 52-year-old female with a history of SLE and APS for 17 years, diabetes mellitus and hypertension for 2 years, two episodes of deep vein thrombosis and three episodes of pulmonary thromboembolism presented with a chief complaint of disorientation and delirium. The patient was taking prednisolone 15 mg daily, azathioprine 50 mg three times daily, hydroxychloroquine 200 mg twice daily, Aspirin 80 mg daily, losartan 25 mg twice daily, amlodipine 5 mg daily, insulin and dabigatran 150 mg twice daily since last year due to a heparin induced thrombocytopenia and possible SLE-related severe thrombocytopenia. Dabigatran was discontinued due to non-adherence to therapy two weeks prior to admission. Brain magnetic resonance imaging (MRI) showed left posterior parietal infarction for possible Libman-Sacks endocarditis given her prior history of SLE. TEE showed two nonmobile small sized masses on the tip of anterior (6x5 mm) and posterior (4x4 mm) mitral valve leaflets (MVLs) indicative of Libman-Sacks endocarditis (figure 2). 

During her hospital stay the patient was treated with the abovementioned medication plus prednisolone 30 mg daily and dabigatran 150 mg twice daily. After a week, her clinical status improved and she was discharged with continuation of therapy. However, during the 1 year follow-up, she was admitted twice, once for an incident of disorientation and once due to SLE flair-up. Follow-up TEE was indicative of no vegetations.

**Case 3: **A 24-year-old male presented with seizure to the neurology department. The patient had no history of previous seizure or neurologic diseases. He reported a history of provoked deep vein thrombosis 3 years ago. Due to the hemolytic anemia and maculopapular rashes, he was suspected to have SLE and possible APS. Laboratory results showed positive ANA, anti-dsDNA, low complements and proteinuria. Kidney biopsy showed stage V lupus nephritis. Lupus anticoagulant and β2-glicoprotein Ab-IgG were also positive suggestive of APS. The patient received prednisolone 75 mg daily (1 mg/kg/d) and was a candidate for cyclophosphamide treatment. He was evaluated for dyspnea before treatment and transthoracic echocardiography showed thickening of MVLs tips. TEE showed two small fixed masses on MVLs suggestive of Libman-Sacks endocarditis without any mobile projections. 

The patient was therapeutically anticoagulated with warfarin and discharged on warfarin 5 mg daily with INR goal of 2-3, prednisolone 25 mg daily, hydroxychloroquine 400 mg daily, aspirin 80 mg and losartan 25 mg twice a day. He received first dose of IV cyclophosphamide therapy with scheduled monthly injections for the next five months. Follow-up TEE was indicative of remnant vegetations but no valvular deterioration.

## Discussion

The involvement of heart valves are common cardiac manifestations in primary or secondary APS including thickening, stenosis, regurgitation and vegetation of the valves. Up to 70% of APS patients have at least one valvular abnormality diagnosed on echocardiography such as thickening, stenosis, regurgitation, and vegetations; however, the most important abnormality is LSE, mostly in mitral and aortic valves (5). 

LSE is a strong risk factor for cerebrovascular events including stroke or transient ischemic attack, as well as, cognitive disability in SLE/APS. The involved valves would have dysfunction including regurgitation or stenosis and require valve surgery. These all together increase the mortality in SLE and APS patients (3). 

Studies have shown an increased rate of LSE in cases with longer disease duration, disease activity, positive anti-cardiolipin antibodies, and or having antiphospholipid syndrome (6). Previous studies have reported an increased rate of cerebral embolism as the common complication of LSE in APS patients (7). APS can occur as a primary disorder or secondary to an underlying disease such as SLE or other systemic autoimmune diseases. Also, one study reported more severe valve dysfunction due to LSE in cases with secondary APS compared to APS alone (8).

All four valves could be involved, but mitral valve followed by aortic valve is the most common valves. Libman-Sacks vegetations are usually located at the base, middle, or tip of leaflets, located on the atrial side of the mitral valve or arterial side of the aortic valve, they have variable sizes and shapes and heterogeneous echogenicity (9). Early detection of LSE can help in early proper therapy and prevention of further related complications. TTE and TEE are used for the diagnosis of LSE and should be considered in high risk patients including patients with recent or recurrent thromboembolic events, stroke, seizure or cognitive dysfunction (1, 3). 

The exact mechanism and proper treatment for LSE is not well-established. Treatment of the underlying disease is necessary, but there is no consensus on the efficacy of the glucocorticoid or cytotoxic efficacy on valvular involvement. However, in all patients with vegetation or significant valvular thickening as well as those with thromboembolic events, anticoagulation therapy should be initiated (9). On the other hand, studies have indicated than even on proper anticoagulation therapy, valvular involvement and LSE occur in APS and SLE patients (10). As one of our patients had LSE while being treated with rivaroxaban. Surgical treatment including replacement or repair should be considered for severe valve regurgitation and stenosis.

In conclusion, we report that in all patients with SLE and secondary APS, TEE should be performed with any evidence of neurologic involvement for early diagnosis and proper treatment of LSE. 
